# Editorial: Nanomedicine targeting central nervous system

**DOI:** 10.3389/fmed.2026.1755137

**Published:** 2026-02-03

**Authors:** Tara Chand Yadav, Barkha J. Yadav-Samudrala, Sylvia Fitting

**Affiliations:** 1 Division of Hematology and Medical Oncology, Department of Medicine, Ellis Fischel Cancer Center, University of Missouri, Columbia, MO, United States; 2 Department of Psychology and Neuroscience, University of North Carolina at Chapel Hill, Chapel Hill, NC, United States

**Keywords:** Alzheimer's disease (AD), artificial intelligence, central nervous system (CNS), drug delivery systems, exosome

In recent years, substantial rise in nervous system disorders have been observed due to lack of early diagnosis and ineffective delivery of drugs to their intended target site. One of the major obstacles to successful treatment is the limited penetration of drugs into the brain, a challenge imposed by the blood-brain barrier (BBB). Although the BBB plays a critical protective role, it also significantly restricts the passage of many drugs and bioactive molecules. To circumvent this, innovative drug-delivery strategies have emerged, transforming the landscape of brain targeting through nanoscale-engineered formulations. Nanomedicines in form of quantum dots, gold nanoparticles, polymeric system, liposomes and polymer-drug conjugates are being extensively investigated for therapeutic applications in high-grade gliomas, neurodegenerative disorders and for diagnostic applications as contrast agents.

Conventional therapies have largely failed to effectively address life-threatening and terminal brain disorders. The emergence of functionalized nanomaterials such as nanosuspensions, bio-nanosystems, engineered macrophages, natural-killer cell, extracellular vesicles, and liposome mediated delivery systems have transformed the field of brain-targeted drug delivery. These advanced platforms have enhanced BBB permeability and improved pharmacokinetic profiles including bioavailability and distribution ultimately restoring synaptic integrity and neuronal repair. It is evident that the future of nanodrug delivery systems is promising, and with continued innovation, the field is poised to make groundbreaking discoveries that will reform and redefine nanomedicine-based targeting of brain cancers and other neurodegenerative disorders.

This Research Topic highlights correlative and synergistic nanomedicine-based strategies that collectively advance CNS disease diagnosis and drug delivery, laying emphasis on therapeutic precision using cutting-edge breakthroughs. Furthermore, the articles in this issue addresses critical challenges such as BBB penetration, disease-specific targeting, sustained efficacy, and translational adaptability, scalability and sustainability, while also identifying existing gaps and emerging needs.

1. *Recent developments in intranasal drug delivery of nanomedicines for the treatment of neuropsychiatric disorders*
  This study explores the recent advances in intranasal drug delivery and focuses on the cutting-edge nose-to-brain (N2B) approach, its advantages over conventional therapy, and its limitations (Kisku et al.). The manuscript underscores the necessity of prolonged therapeutic interventions in cases of neuropsychiatric conditions and how the application of nanomedicine-based targeted drug delivery offers a compelling solution to facilitate site-directed, sustained-drug release, improved pharmacokinetics and enhanced BBB penetration efficiency and efficacy for CNS disorders. Apart from discussing technical advances, this article expands current understanding of CNS drug delivery by evaluating formulation strategies designed to achieve sustained release, improve pharmacokinetics, and extend therapeutic residence time in the brain. Importantly, it also raises timely questions regarding the long-term safety and clinical translatability of intranasal nanoformulations for the treatment of neuropsychiatric disorders.
2. *Recent advances in nanotechnology for Parkinson's disease: diagnosis, treatment, and future perspectives*
  This manuscript evaluates nanotechnology-based approaches for the management of Parkinson's disease (Yadav et al.). It underlines the limitations of current treatment options available to curb and treat Parkinson's symptoms, including surgical methods and receptor-based pharmacological therapies using small molecules. The emphasis is laid on employing nanocarriers like carbon nanotubes, metallic nanoparticles, polymeric and lipid-based nanoparticles, including dendrimers and solid-lipid nanoparticles, to improve therapeutic delivery and achieve better clinical outcomes. This article draws attention to emerging disease-specific nanocarriers and their ability to overcome the limitations of receptor-based therapies and invasive surgical treatment procedures. It further emphasizes the integration of diagnostic and therapeutic interventions to aid early disease prediction and precision-guided therapy, while underscoring the need for longitudinal *in vivo* validation and patient-specific drug delivery approaches.
3. *Targeted drug delivery in neurodegenerative diseases: the role of nanotechnology*
  This review article briefly summarizes the potential of nanotechnology-based platforms for neurodegenerative disorders using nanotherapeutics (Dhariwal et al.). It highlights how advanced nanomedicine approaches can enhance penetration through the BBB and improve transport of therapeutic agents such as GM-CSF to disease-relevant sites. These strategies offer promising avenues to overcome current challenges associated with the treatment of progressive neurodegenerative disorders, such as Huntington's, Alzheimer's, and Parkinson's diseases. The salient features discussed in this article revolves around immune-modulatory cargo delivery and its potential to support disease-modifying, rather than merely symptomatic, therapeutic strategies, while prompting future investigations into combinatorial nanoformulations and smart, responsive delivery systems tailored to disease exacerbation and progresssion.
4. *Artificial intelligence-based advancements in nanomedicine for brain disorder management: an updated narrative review*
  This narrative systematic review analysis underscores the imperative need for machine learning, deep learning and artificial intelligence to modulate nanomedicine therapy (Dipankar et al.). It offers critical insights into the design and fabrication of nanomedicine using computational methods to accelerate disease diagnosis and biomarker identification for better management of brain cancer, as well as neurodegenerative disorders, including multiple sclerosis, Parkinson's disease, and Alzheimer's disease. This article meaningfully extends the emerging role of artificial intelligence (AI) beyond formulation design, positioning AI as an integrative platform for biomarker discovery, predictive modeling, and optimization of therapeutic interventions. It further highlights critical future needs for data standardization, regulatory frameworks, and real-world clinical integration of AI-guided nanotherapeutic interventions.
5. *Therapeutic and diagnostic implications of exosomes as natural nanoparticles: a new paradigm in brain cancer disease management*
  Exosomes are extracellular vesicles capable of delivering drugs to target sites and are being comprehensibly explored to target the brain to improve the therapeutic efficiency of drugs, especially in brain cancers, due to their endogenous origin, excellent biocompatibility, high drug-loading capacity, and BBB penetration potential (Mohiyuddin et al.). This review article summarizes the enhanced efficiency of drug-loaded/functionalized exosomes in crossing the BBB, their efficient cellular uptake, and their remarkable ability to treat brain cancer. It also explores their applications in disease diagnosis and theragnostics. The key strength of this article is its emphasis on the shift from synthetic to endogenous, cell-based delivery carriers with theragnostic potential, while drawing attention to strategies for enhancing BBB penetration and minimizing immunogenic responses. The article also acknowledges unresolved challenges related to cargo heterogeneity, regulatory considerations, and scalability, all of which are critical for successful clinical translation.
6. *Nanoparticles: A New Frontier in Neurodegenerative Disease Therapy*
  This review article discusses targeted brain delivery using nanodrug-delivery systems, including polymeric micelles, dendrimers, and liposomes to improve the clinical outcomes in neurodegenerative disorders (Kumar et al.). It also highlights, how fabricated nanodrug delivery platforms result in enhanced bioavailability and biocompatibility, and nanosizing leads to large surface area and offers tunable properties, involving functionalization with small molecules, antibodies, nucleic acids, and peptides, rendering these nano-delivery systems excellent carriers for therapeutic interventions in gene therapy. This article advances the field of gene therapy and multifunctional, immune-compatible nanoplatforms, thereby extending the scope of nanomedicine beyond conventional small-molecule delivery. It also outlines future directions and emerging trends, including tailored functionalization, controlled regulation of gene expression, and comprehensive biosafety evaluations.

Collectively, these articles demonstrate the evolving landscape of nanomedicine and provide a proof-of-concept drug delivery approach to integrate smart and biologically informed platforms capable of transforming CNS disease diagnosis, mitigation and treatment. This Research Topic also underscores a paradigm shift in brain-targeting using nanomedicine using computationally optimized therapeutic systems to circumvent physical delivery barriers and lead to biologically responsive precision-medicine. Future research needs to be more focused on prioritizing the enhancement of safety and efficacy profile, increasing the scalability and regulatory standardization of advanced nanocarriers, and integration of artificial intelligence to render personalized therapy, real-time disease modeling and treatment optimization. Tackling these incumbent challenges using interdisciplinary teamwork will be crucial to translating these nanomedicine-based interventions from bench to clinically viable solutions with positive effects on patient care.

